# Enabling activity in palliative care: focus groups among occupational therapists

**DOI:** 10.1186/s12904-019-0394-9

**Published:** 2019-02-07

**Authors:** Sofia Tavemark, Liselotte N. Hermansson, Karin Blomberg

**Affiliations:** 1 Faculty of Medicine and Health, School of Health Sciences, Örebro University, S-70182 Örebro, Sweden; 2 Örebro Municipality, Healthcare and Social Services, Örebro, Sweden; 3 University Health Care Research Center, Faculty of Medicine and Health, Örebro University, Örebro, Sweden

**Keywords:** Activities of daily living, Leisure activities, Client participation, Quality of life, Qualitative research

## Abstract

**Background:**

Activity participation may support clients in palliative care to maintain dignity and quality of life. Literature and policy documents state that occupational therapists should be part of the team in palliative care but provide limited guidance on how interventions should be employed. Thus, the aim was to describe occupational therapists’ experiences of enabling activity for seriously ill and dying clients.

**Methods:**

In a descriptive, qualitative study, three focus groups with occupational therapists (*n* = 14) were conducted. The data were analysed using qualitative content analysis.

**Results:**

The findings showed that occupational therapists enabled activity in clients in palliative care while considering the client’s individual preferences. Motivation was seen to facilitate activity, while environmental restrictions were thought to act as barriers. The occupational therapists wanted to bring activities physically closer to the clients and felt a need for more client contact to enable activity.

**Conclusions:**

Occupational therapists’ interventions in palliative care include prioritizing and planning activities according to clients’ preferences and capacities. The individual nature of these activities makes it impossible to create standardized protocol for interventions, but the study results can be used to describe occupational therapists’ strategies and to guide their work, especially for occupational therapists without experience in palliative care.

**Electronic supplementary material:**

The online version of this article (10.1186/s12904-019-0394-9) contains supplementary material, which is available to authorized users.

## Background

Palliative care aims to improve the quality of life of clients facing life-threatening illness and of their families [1]. Palliative care should be provided regardless of diagnosis or age and should not only include patients in the late phase of the end of life. Instead, palliative care should be considered earlier in patients with serious illnesses. Palliative care is not only provided by specialized palliative clinics but also in general care facilities, such as community-based care facilities including nursing and care homes or through home-care [2]. It has been proposed that care should be based on a holistic perspective, which requires a supportive care environment in which non-medical interventions, such as meaningful activities, are of great importance for quality of life [2–4]. Enabling meaningful activities is a way to enhance quality of life and support clients to live with dignity despite a reduced ability to be active. Activity can be described as a person carrying out a task or an action [5]. Hence, regardless of the client’s stage of life, occupational therapists should support clients to find strategies to deal with social isolation [6], to redefine their roles [7], and to enable activity by adjusting the clients’ environments. Participation in activity may help clients to maintain their identity [7] and their self-esteem [6, 8]. Positioning can help clients to improve their comfort [4, 9] and, with strategies to limit exertion [10], they can continue to carry out the desired activities. These efforts, together with relaxation [11, 12] and reminiscence, help clients to dispel thoughts that can act as barriers [4, 6, 13] and to deal with anxiety and pain [12]. In the final stages of life, the ability to perform activities is affected in various ways. This ability may depend on clients’ wishes, habits, and capacity to be active [7]. Many clients in palliative care wish to have an influence over their care [8, 9, 14–17] and to have control over their daily lives [6, 17, 18]. Earlier studies have described that this can be achieved through participation in meaningful activities [4, 6, 10, 15, 19] in which the client and the occupational therapist together plan activity-related goals [10, 15, 16, 20–22]. It is important for clients to find the motivation to be active [10] and to feel valued [6, 8]. Clients often want to maintain or improve their independence [17, 22, 23], physical function [16, 24], and activities of daily living [10, 17, 22–24] in order to live as normally as possible [3, 11, 16, 17, 22]. They want to be able to spend time with their loved ones [4, 6, 11, 16, 17, 19, 21] and possibly to be able to return home [22]. They also want meaningful activities to fill the day [6, 7, 19] and to feel hope [20]. In Sweden, the occupational therapist is described as part of the team that works with clients in palliative care [25]; however, the literature and policy documents provide limited guidance on appropriate non-medical evidence-based interventions [3] compared to medical and nursing recommendations in palliative care that have been well described. Additionally, studies state that more practice and support needs to be available to prepare occupational therapists to work with palliative care clients [26, 27]. To summarize, existing research describes the importance of working with activities to relieve symptoms, enable social relationships, and improve the client’s self-image, self-determination, and situation [4, 6, 13, 22, 23], but this research does not make clear what those activities are or how to enable them. By describing how activities are used in palliative care by capturing the expert opinions of those already working in the area, new understanding and knowledge can be generated. An increased understanding of how to enable activity in the final stages of life can clarify the function of the occupational therapist in palliative care, and finally the care can be improved, with a focus on increased quality of life and maintaining dignity for seriously ill clients. Thus, the aim of the study was to describe occupational therapists’ experiences of enabling activity among clients in palliative care.

## Methods

### Design

The study had a descriptive design with a qualitative approach [28]. The data consisted of focus group discussions (FGDs), as these data collection methods aim to facilitate individuals’ exploration and clarification of their views through group interaction in a manner not possible in one-to-one interviews [29]. Ethical approval for the study was obtained from the Regional Ethical Review Board of Uppsala, Sweden (reg.no: 2014/312).

### Participants

**Fig. 1:**
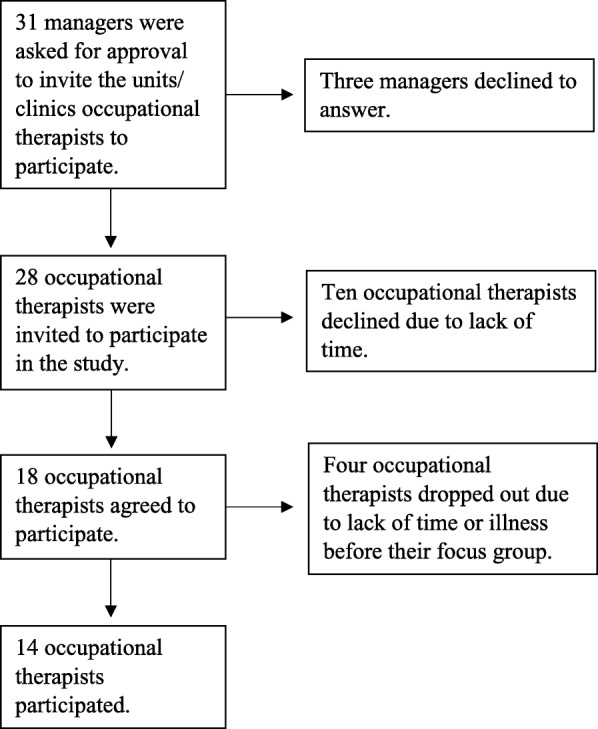
Recruitment process

The community-based palliative care coordinator and the managers at the University Hospital clinics in the middle of Sweden who regularly care for clients in the final stages of life were asked to identify participants (Fig. 1). Through strategic sampling [28], occupational therapists with varying experience levels were recruited. Various care contexts were included to cover multiple perspectives on the topic and to reduce the risk of missing important information [29]. The goal was to find participants from community-based home-care services, community nursing and care homes, and hospital based healthcare, taking into account (i) the length of professional experience, (ii) sex, and (iii) age distribution. The inclusion criterion was being in regular contact with adult clients undergoing palliative care, in either the early or late palliative phase.

Potential participants were sent an information letter via email. If there was no response, two reminders were sent at weekly intervals. Those who agreed to participate gave written informed consent and chose between different prearranged times for the focus group. A total of 14 occupational therapists participated (for demographic information, see Table 1).

**Table 1:** Participant characteristics (*n* = 14)

	n
Male	1
Female	13
Age	
33–41	2
41–49	2
49–57	4
57–64	6
Experience of occupational therapy practice at present (2014) ^a^	
1–12 years	4
12–23 years	4
23–34 years	1
34–44 years	4
Experience of palliative care ^a^	
1–5 years	1
5–10 years	5
10–15 years	4
15–25 years	3
Healthcare context ^a^	
Urban home-care services	5
Rural home-care services	0
Nursing and care homes	5
County council clinics	4
Course in palliative care ^a^	
Yes	10

^a^data from one subject missing

### Data collection

The participants from the different care contexts were divided into three focus groups of three, five, and six participants, respectively. This was done to stimulate discussion and to capture the experiences of different healthcare contexts [29]. The focus group discussions were held in Swedish, moderated by the first author (ST) and conducted during the participants’ working hours in an activity room at the hospital. The moderator’s role was to facilitate the discussion and focus on the following topic: “*Please describe how you enable activity for clients in palliative care.*” The moderator also used follow-up questions (see Additional file 1), brought the topic back into focus when the group digressed from the topic, and ensured that the quieter participants had a chance to speak. All focus groups began with the moderator welcoming everyone and providing overall information on how the discussion would be conducted. Thereafter, the participants shared their experiences of palliative care before the predetermined questions were used as a basis for the discussion. An observer attended and made notes on the basis of the discussion to indicate which topics participants were most engaged in and where their opinions differed. The notes were used as support when the moderator summarized the discussion. The first focus group was used as a pilot group. No changes to the procedure were necessary. The pilot focus group was therefore included in the analysis. The discussions went on for 60 to 90 min and were audio recorded. All recordings were transcribed verbatim.

### Data analysis

The focus group discussions were analysed by using inductive qualitative content analysis [28, 30], focusing on the manifest content. First, the transcripts were read through several times to obtain an overall picture of the data, and meaning units that corresponded to the research aim were identified. They were then condensed to make the text more manageable without losing the content. The condensed meaning units were coded and grouped into subcategories according to content. Categories were formed on the basis of similarities and differences in the subcategories to reflect the experiences of occupational therapists working in palliative care (see Table 2). All authors read individually the transcribed discussions and compared the codes and categories to increase trustworthiness. Quotations have been used to illustrate the results [28].

**Table 2:** Example of the analysis process

Meaning unit	Condensed meaning unit	Code	Subcategory	Category
And all the trips that are made too, I know when I was out there and we ... I, I still have an image in my head, we were out, now I can’t remember how, but it was in the final stages for those gentlemen that we took out to [place name], and they are sitting on a jetty with fishing rods with no hooks on but and smoking ci- ci- cigars, both those two gentlemen, it was kind of this quality of life.	Going on requested excursions at the end of life to have quality of life	Excursions that provide quality of life	Activity that provides quality of life	Activities based on the client’s wishes and possibilities
But of course you notice this, we’ve got this personal, personal integrity, yes, that we talk about and you notice this of course, that they really want to manage this, especially going to the toilet by themselves, and they would like to manage all the personal things ... uh, if I’m to go and make an ADL assessment and that sort of thing, then I can, but of course I feel that it can feel awkward because they think they can feel ashamed that they can’t manage that particular thing themselves and that they want to manage all that, eating and drinking themselves, and going to the toilet and dressing themselves and taking care of their personal hygiene.	Dignity means that the person wants to manage personal tasks independently	Personal activities to maintain dignity	Activity to maintain dignity	

## Results

The FGDs allowed the participants to confirm and to elaborate on each other’s arguments. All participants agreed on the importance of activity for clients in palliative care. They all worked with activities in a similar way regardless of the care context. Their experience showed that they focused on enabling different activities depending on the client’s wishes and current environment. Five categories were identified by the analysis: “enabling activities based on the client’s wishes and circumstances”, “prioritizing activities together with the client”, “facilitating factors to enable activity for clients in palliative care”, “limiting factors to enable activity for clients in palliative care” and “areas for improvement to enable activity for clients in palliative care” (see Table 3).

**Table 3:** Categories and sub-categories from the results

Categories	Activities based on the client’s wishes and circumstances	Prioritizing activities together with the client	Facilitating factors to enable activity for clients in palliative care	Limiting factors to enable activity for clients in palliative care	Areas for improvement to enable activity
Subcategories	Activity to get away from the care environment Important to return home Activities that require little energy Activity for reflection and support Activity to maintain physical ability Fulfilling responsibilities Conversation Carrying on as usual Being included in activities Planning the activity themselves Activity to maintain integrity Participating in group activities Individual activity Activities that give quality of life Not wanting or managing activity	Activity depending on the client’s wishes and motivation Focusing on remaining health through activity Customizing activities to the person and context The client’s physical ability influences the choice of activity Activity to allow social interaction Taking relatives into account when planning activity	A client with a positive attitude and motivation Clients with good communication skills A team that is in consensus Physical factors in the environment	Limitations in the physical environment Lack of cooperation Establishing contact at a late phase Lack of resources Clients with a negative attitude or impaired insight Lack of communication with the client The client’s physical limitations due to illness Staff who follow written procedures rather than being responsive to the client’s needs Clients with a negative attitude to aids Negative consequences of activity	Need nearby premises for activities Need to talk more Need more time for activities Find new ways to bring clients together for activities

### Enabling activities based on the client’s wishes and circumstances

The occupational therapists pointed out that it is important that they have regular contact with clients and find ways to communicate that enable self-determination. They guide the clients so that the clients can plan their activities themselves. The participants described how they enable activity by informing, prescribing and instructing clients in the use of aids, making adaptations in the home, conversing, adjusting strategies to facilitate activity, and assessing what level of help the clients need to make activity possible for them.

The occupational therapists described that they support clients to return home to their normal environment. They talked about the clients’ desire to get away from the care situation in order to increase their quality of life and make time for reflection, for example, by visiting previously familiar environments or spending time outdoors, regardless of the care context. Conversations about living and dying in various forms were described as the most requested activity.

The FGDs showed that there is a difference in regularly offered activities depending on the care context. Occupational therapists in nursing homes offered more group activities due to accessible environments than therapists in other facilities. In home-care situations, the focus was on maintaining clients’ daily lives within their homes, and in hospital care situations, clients were offered individual activities that required limited energy expenditure. However, the participants’ common experience was that many clients devoted most of their energy to performing personal tasks as independently as possible for as long as possible to maintain their dignity, particularly with regard to using the toilet. The occupational therapists stressed the individual nature of clients’ preferences for group vs individual activities. According to the participants’ experiences, clients often wanted to continue with the activities that they had previously been involved in, such as cooking, religious activities, gym exercises, or music, as a strategy to relieve their worries and anxiety. The occupational therapists’ experience was also that some clients wanted to honour their commitments to reduce inconvenience for their families and to be able to rest assured at the end of life.

Occupational therapist 9: “*We had a man who summoned his energy because he had to get out and sell his car so that his wife would not have to trouble herself with that. Many are worried about their relatives, about what will happen, and such things. So he really made an effort, and then he was off and dealt with getting rid of the car. It was like he had it on his mind for a few weeks: that it was an important thing for him to get done.*”

Occupational therapist 8: “*Most of the time, it is the older generation of men who have had the responsibility for everything. And as you say, nobody else should be troubled with that.*” (Focus group 2)

The participants described that it is important for many clients in palliative care to be offered activities that are not energy intensive, such as playing cards, painting, doing needlework, reading, or solving crossword puzzles. Sometimes clients do not wish to participate in any activity, and then the occupational therapist must focus on symptom alleviation interventions, allowing the client to lie comfortably or to listen to music to enable rest.

### Prioritizing activities together with the client

In the FGDs, it was stressed that the occupational therapist should be one step ahead and constantly attuned to the surroundings. The common experience was that compensatory interventions are applied at an earlier stage in palliative care than in regular care to enable activity instead of restorative training to overcome the obstacle.

Occupational therapist 2: “*I think that I probably enable it in that I’m fast, so to say. If it is about an aid to be able to enable an activity, an office chair to allow someone to be active… you give it priority, and you take that step before you might have done it for a different client. In that case, you would have waited, … but it’s not like that in this case; here, you don’t hesitate*.”

Occupational therapist 6*:* “*It’s a good thing when seriously ill clients have a request; if they want to come home, we have to act right now because in two days, it may be too late.*” (Focus group 1)

A common experience was that activities are prioritized together with the clients, depending on their capacity for activity, regardless of the type of care provision. Each client’s motivation, desire, and experience of meaningful activities affects the choice of activity. The participants emphasized that their approach, namely, continuing to allow clients to be active according to their wishes as long as possible, is important when activities are prioritized. The occupational therapist must be sensitive and strive to get to know the client and the circumstances in order to be able to prioritize activities. They made it clear that the context can be crucial for the feasibility of the activity and how the client experiences it. It is therefore important to consider and involve relatives when planning activities.

Occupational therapist 8: “*Sometimes relatives come, and it can be surprising because then it can suddenly be things that can appeal to them; it gains a context. Unwinding in front of the TV with the family on Friday nights is one such thing, which can consist of a bit of this and that, like crisps and dip, and, yes, a little wine and all that, and it can be appealing, you know*.” (Focus group 2)

### Facilitating factors to enable activity for clients in palliative care

One of the main factors that facilitates activity regardless of the care context is the clients’ attitudes, which are influenced by wishes and motivation. It helps if clients have a positive attitude to aids, if former acquaintances and important places are still there to visit, or if premises are available where activities can be performed when it suits the clients without requiring too much coordination. The occupational therapists who worked in home-care situations stressed that a well-functioning team is beneficial because it is an important source of information for them. The teams that work towards a common goal and use their knowledge and available resources help to facilitate activity. All participants agreed that committed relatives are an important factor in enabling activity, and relatives themselves need to be supported if the client is to continue to be active. They mentioned the need for a well-functioning organization in healthcare so that time can be given to each client and activities can be performed promptly. It is also important that collaboration within and outside the county borders works well to be able to satisfy wishes that cross county boundaries. It helps if the organization has procedures for how knowledge of the clients’ wishes should be managed and if there are flexible staff members who can give the time clients need.

### Limiting factors to enable activity for clients in palliative care

Regardless of the care context, lack of financial resources and time were seen as barriers to enabling activity. The occupational therapists noted that they are responsible for a large number of clients and must decide which interventions should be implemented, taking cost-effectiveness into account. Lack of time can also lead to the healthcare staff working to a routine, which reduces the client’s opportunities for self-determination. The experiences show that cooperation may be lacking at multiple levels including as a result of unclear boundaries between different organizations and fixed views about who is responsible for what. The range of activities offered is sometimes influenced by what the staff members are comfortable with or by personal values among the staff, which indicates that they are not always sensitive to the client’s wishes. The participants also described that cooperation with relatives can fail if the relatives have no insight into the client’s situation, which can lead to difficulties in understanding the trade-off between benefits and possible consequences of activities. Psychological factors among clients, such as reduced motivation and attitude, can affect the performance of activities. They further reported that certain clients are afraid that their home will look similar to a healthcare environment or that the client’s identity will be affected, making it difficult for the clients to accept the use of assistive technology in order to enable activity.

Occupational therapist 6: “*... she doesn’t want to accept it: ‘I'm not old, I don’t want to be handicapped, and these people will of course laugh at me’.*” (Focus group 1)

The occupational therapists explained that the client’s health status, including conditions such as nausea, pain, or fatigue, limits the range of activities. This gives rise to a dilemma about when clients should be given priority but symptoms take time away from other activities for the client. The participants also described that a lack of communication skills in clients can be a major difficulty when planning interventions. Furthermore, it is difficult to interpret the client’s wishes in the late palliative phase and if the client is unfamiliar to the occupational therapist.

### Areas for improvement to enable activity

The participants highlighted the need for more nearby, available premises for activities and more ways of bringing activities closer to the clients, e.g., for in-hospital care they wished for a trolley with games and facilities for painting, needlework, reading, and music. The occupational therapists in nursing and care homes stressed their wish for the opportunity to transport the client between the home and the activity as an improvement. Regardless of the care context, the participants suggested that there is a need to talk more, partly by letting the client express their perceived participation in their healthcare, and partly through discussion groups, where clients and their families would be able to share their experiences. Most participants from home-care and hospital care situations wanted more client time and less time working with administrative procedures. All participants argued that the team must also become better at making time to give a client golden moments in their everyday life. One area for improvement was to become better at doing things together with the clients and their relatives. The occupational therapists emphasized the importance of ensuring that staff with knowledge of the clients and the clients’ capacity should be the people responsible for arranging the activities, in order to be able to offer high quality activities to the client.

## Discussion

The absence of occupational therapy in relevant health policy documents is a documented problem in several countries. The Canadian Association of Occupational Therapists (CAOT) (31) and Occupational Therapy Australia (OTA) (32) argue that occupational therapy is important in palliative care to assist clients to live safely and comfortably in the place of care of their choice. The CAOT and OTA (31, 32) state that the role of occupational therapists should be outlined in health policy documents to enhance knowledge about occupational therapy in palliative care. The results from this study may serve as a contribution for the development of future guidelines. The findings in this study identify occupational therapists’ experiences of enabling activity for clients in palliative care and thus clarify the function of occupational therapy in palliative care and provide a benchmark for interventions. This is especially important as detailed description of appropriate occupational therapy interventions in palliative care is lacking [2, 31, 32]. Participants emphasized that activities should be based on the client’s wishes and circumstances and prioritized according to the client’s motivation and ability. Previous studies have highlighted that it is the client’s wishes that determine which activities are prioritized [15, 20, 33], which requires that staff with the right knowledge support the client during these activities. This finding is consistent with those of Costa and Othero [7], who pointed out that occupational therapists’ salutogenic, holistic approach and knowledge of physical and cognitive abilities is suited to helping clients to have the best chances for performing activities. The occupational therapists in this study pointed out that while some clients just want to lie comfortably in bed, others want a broad spectrum of activities. The desired activities described range from those that are not energy intensive to everyday personal tasks to excursions of various kinds, which is consistent with earlier research [17]. The occupational therapist aims to maintain client stability in daily life in the context of the ongoing change that illness brings. The participants stressed that many clients wish to continue as usual, which has also been found in other studies [3, 11, 16, 17]. Park, Lala and Kinsella [6] argue that activities are a form of reflection and a way to experience meaning at the end of life. An understanding of clients’ varying wishes places demands on the occupational therapist to offer individually tailored occupational therapy. The study findings highlight the importance of the relationship and communication between the client and occupational therapist to enable care. The conversations may cover existential issues, and some clients, but not all, may want to talk about dying. Participants believed that an established relationship makes it easier for the occupational therapist to offer appropriate activities in the final stages of life. Hence, it is problematic when the occupational therapist does not establish contact until the late palliative phase, which was seen by the participants as a limiting factor for enabling activity in palliative care. This may partly be due to the team’s lack of knowledge about the occupational therapist’s role in palliative care [14]. It is also important that the occupational therapist help clients adapt old habits and make activities available to them [7]. Several participants felt that they had to give low priority to other clients who were not in palliative care in order to make the necessary time available for clients in palliative care.

In palliative care, it is important that clients maintain their identity and self-image [10, 16, 17]. Participants noted that clients are sometimes afraid of losing their identity or self-image. Both Costa and Othero [7] and Eriksson, Öster and Lindberg [17] argued that the occupational therapist can be helpful when clients need to reflect on and adapt their roles in everyday life. This study highlights the importance of the occupational therapist in communicating with the team when the care of the client is being planned and in working together with the client to plan everyday activities. This was described as a factor that facilitates activity in palliative care.

The results also illustrate clients’ increased needs for advanced medical care, which in turn emphasizes activities, such as recreation and reflection. The participants in this study believed that there are greater demands on the occupational therapist in palliative care to make activities available and to help the client to adopt new habits. It has also been suggested in a previous study that occupational therapy in palliative care needs to evolve from focusing on home adaptations and trying out aids to include a wider repertoire of activities [14]. This study not only shows the importance of aids and home adaptions but also highlights the dilemma between aids and social support. The occupational therapists pointed out areas for improvement to enable activity, such as wishes to spend more time with clients and their relatives to increase quality in palliative care through psychological support and to talk about activities with clients and their relatives.

Rehabilitation and the occupational therapist’s role in palliative care have been described earlier [34]. It is already known that the occupational therapist has a large burden of non-client-related tasks [33]. The participants in this study came to the conclusion that they can focus more on the client and enable activity by offering more individual time with the client, which is in line with the findings of Eriksson, Öster and Lindbergs [17] and by finding ways to bring activities closer to the clients. However, they felt that occupational therapists need more organizational support to allow them to spend more individual time with the clients and adapted premises to enable them to offer clients in palliative care high-quality individual activities, a concept previously highlighted in the literature [8]. However, activities may not be the best intervention if the client is not motivated. Krishna, Yong and Koh stated that palliative rehabilitation is not effective for all clients and must be seen from a holistic perspective [21]. Future research should examine the team members’ knowledge about the occupational therapist’s role in the multidisciplinary team in order to alert the group as to when occupational therapy is needed. It would also be worthwhile to investigate clients’ views on the activities they are offered in order to provide activities optimally and at the appropriate time in the final stages of life.

### Methodological considerations

This study has some potential weaknesses. The size of the focus groups was uneven, and there were few participants in each group. Small focus groups pose the risk of losing the total breadth of experiences and can make the results difficult to implement in other care contexts. However, the risk of this was low in this study due to sampling [29] since several care contexts were represented in all groups, which enriched the discussions. The participants were acquainted with each other without knowing each other well, which meant that they could give each other more perspectives on the topics and, as the interaction was dynamic, openly discuss the questions [35]. Focus group discussions obviously provide rich data and are an appropriate method for collective insight [29]; the occupational therapists in this study interacted and built upon each other’s experiences and interpretations. However, having smaller focus groups can also be seen as advantageous because a group with fewer participants is easier for an inexperienced moderator to handle, and the participants will be easier to distinguish in the audio recordings [29]. The low number of participants also allowed everyone to make their opinion heard and ensured good group dynamics. There is always a risk that the credibility of a qualitative study is threatened when using an inexperienced moderator [28]; inexperience may affect the moderator’s ability to ask follow-up questions and stimulate the discussion [29]. Nevertheless, the credibility was strengthened by the fact that the moderator used predetermined questions to ensure that several perspectives on the topic were discussed and asked follow-up questions to confirm that the participants’ statements had been understood correctly. Furthermore, the moderator and the observer met directly after each focus group to ensure that they had interpreted the discussions in the same way. The subject of the discussions also stimulated the participants to be active, which meant that the discussions went well; however, other perspectives may have existed but been overlooked in the discussions. The credibility of the study was strengthened by the inclusion of participants with relevant experience, some having worked many years in palliative care, and by the fact that all the focus group discussions took place within one week. We cannot exclude the risk that the moderator influenced the participants or that some participants at times steered the discussions. There is also a risk that the participants interpreted the moderator’s questions in different ways or that they wanted to give the “correct answer” [29]. For this reason, the moderator pointed out to the participants at the beginning that there were no right or wrong answers and that all experiences were equally important. The moderator’s role also allowed participants to associate freely beyond the predetermined discussion questions and the other participants’ statements, which enriched the discussions.

## Conclusions

Occupational therapists must tailor their work to meet clients’ individual wishes in palliative care. Enabling activity includes acquiring an overview of which activities clients usually perform and helping them to prioritize and plan activities according to their preferences and capacities as a person. Strategies need to be described in the relevant policy documents and in clinical guidelines to guide occupational therapists to create evidence-based recommendations and to ensure that the entire team understands the role of occupational therapists in palliative care. In this way, we can increase our current knowledge of the scope of occupational therapy and ensure that clients can participate in meaningful activities towards the end of their lives. Further studies are needed to increase knowledge about the clients’ own wishes and needs.

## Additional file

Additional file 1: Interview guide. (DOCX 18 kb)

## Abbreviation

FGDs: Focus group discussions
