# Supplementary material for: Enabling activity in palliative care: focus groups among occupational therapists
Source: BMC Palliat Care. 2019 Feb 7;18:17. doi: 10.1186/s12904-019-0394-9 (PMC6367774; doi:10.1186/s12904-019-0394-9)
Supplement: Supplementary file 1 — Interview guide. (DOCX 18 kb) [file 12904_2019_394_MOESM1_ESM.docx]

Interview guide

## Areas for discussion

*Can you tell us how you view activity for clients in palliative care?*

- Please describe how you enable activity for clients in palliative care?
- Can you describe and exemplify what activities you suggest?
- Can you describe a situation where it was ‘easy’ to enable activity?
- Can you describe a situation where it was ‘difficult’ to enable activity?

*Is there anything that can be changed in order to enable activity for clients in palliative care*?

- In your opinion, what would enable these changes?
- Is there anything that prevents these changes from being implemented?

## Follow-up questions:

- What do you mean?
- Can you explain?
- Can you give an example?
- What makes the difference?
